# Supplementary material for: Preclinical and randomized clinical evaluation of the p38α kinase inhibitor neflamapimod for basal forebrain cholinergic degeneration
Source: Nat Commun. 2022 Sep 21;13:5308. doi: 10.1038/s41467-022-32944-3 (PMC9492778; doi:10.1038/s41467-022-32944-3)
Supplement: Supplementary file 1 — Supplementary Information [file 41467_2022_32944_MOESM1_ESM.pdf]

Supplementary Figure 1. Hallucinations Severity Over Time on Study

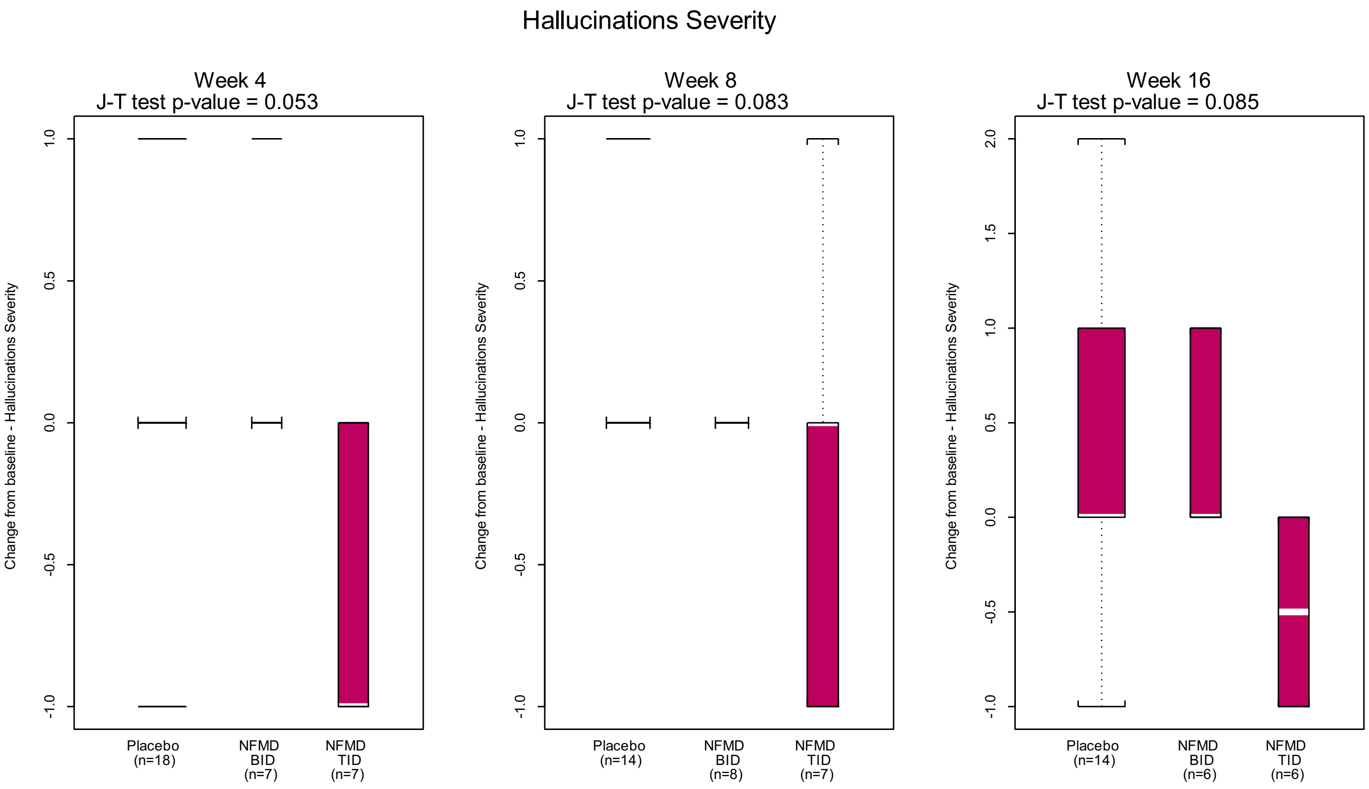

Legend:

Change from baseline [median, intra-quartile range in red, and range] is shown for each domain at each time point. A reduction in score reflects improvement. “J-T test” is the Jonckeere-Terpstra test for dose repones for greater reduction (improvement) in the order of placebo (lowest)<NFMD BID<NFMD TID (greatest amount of reduction).

Supplementary Figure 2. 10-Item Neuropsychiatric Inventory Domains of Interest

Change from baseline [median, intra-quartile range in red, and range] is shown for each domain at each time point. A reduction in score reflects improvement. "J-T test" is the Jonckeere-Terpstra test for dose repones for greater reduction (improvement) in the order of placebo (lowest)<NFMD BID<NFMD TID (greatest amount of reduction). "FxS Score" = frequency times severity score (range 0-12).

Hallucinations

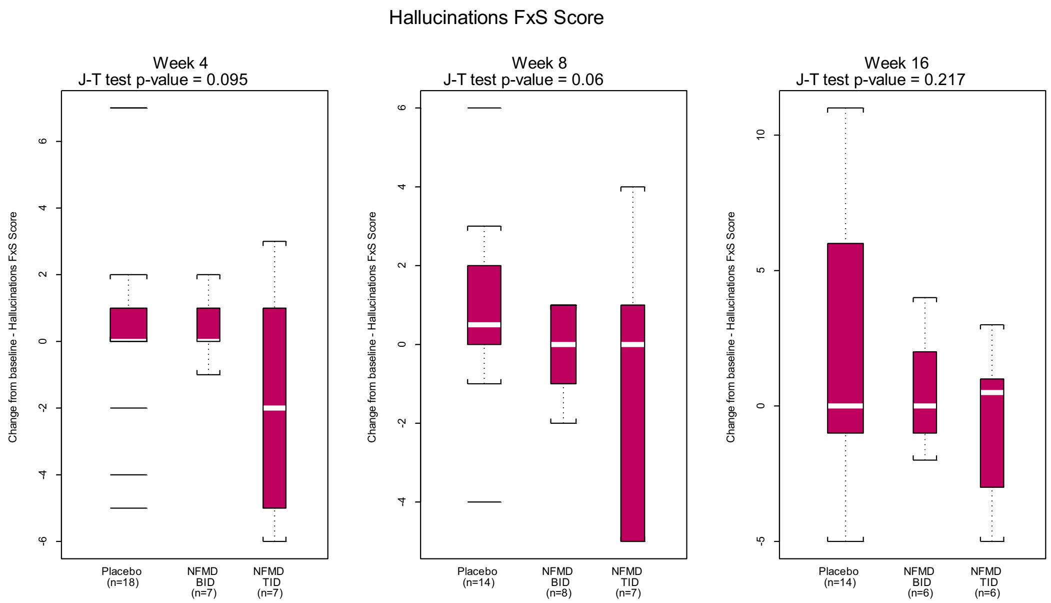

Agitation/Aggression

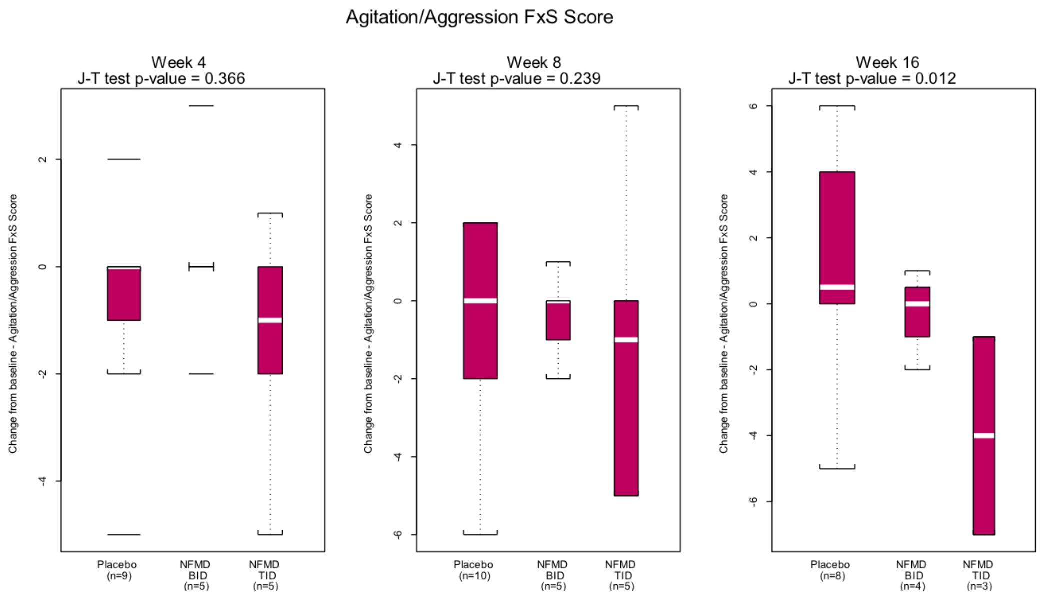

Depression/Dysphoria

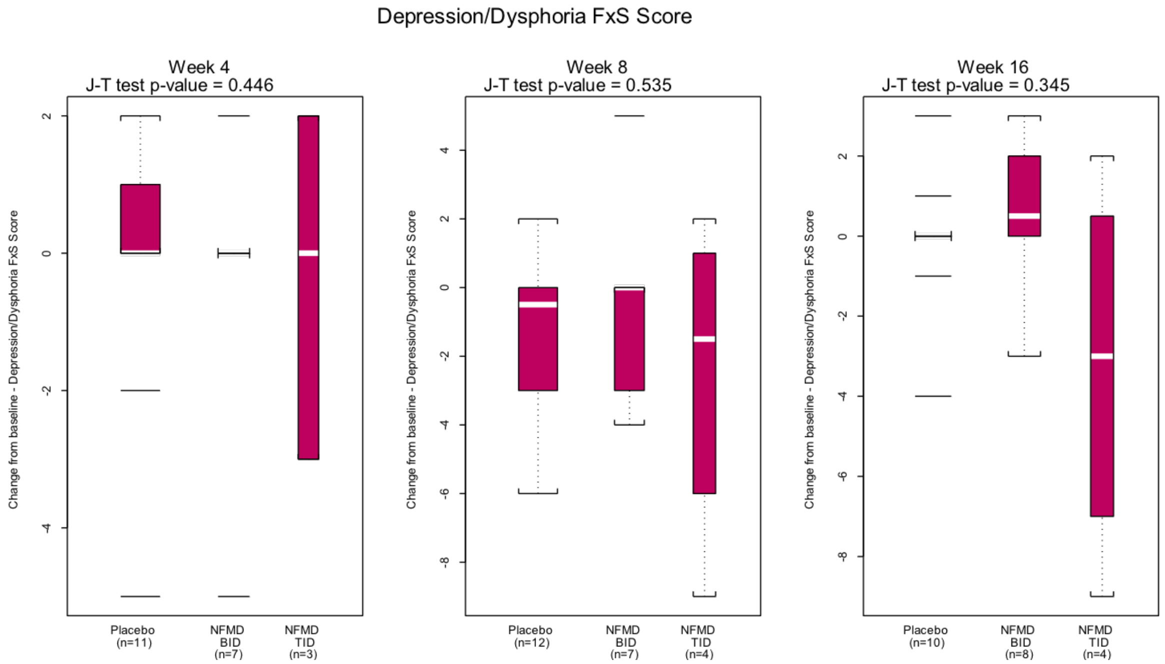

Anxiety

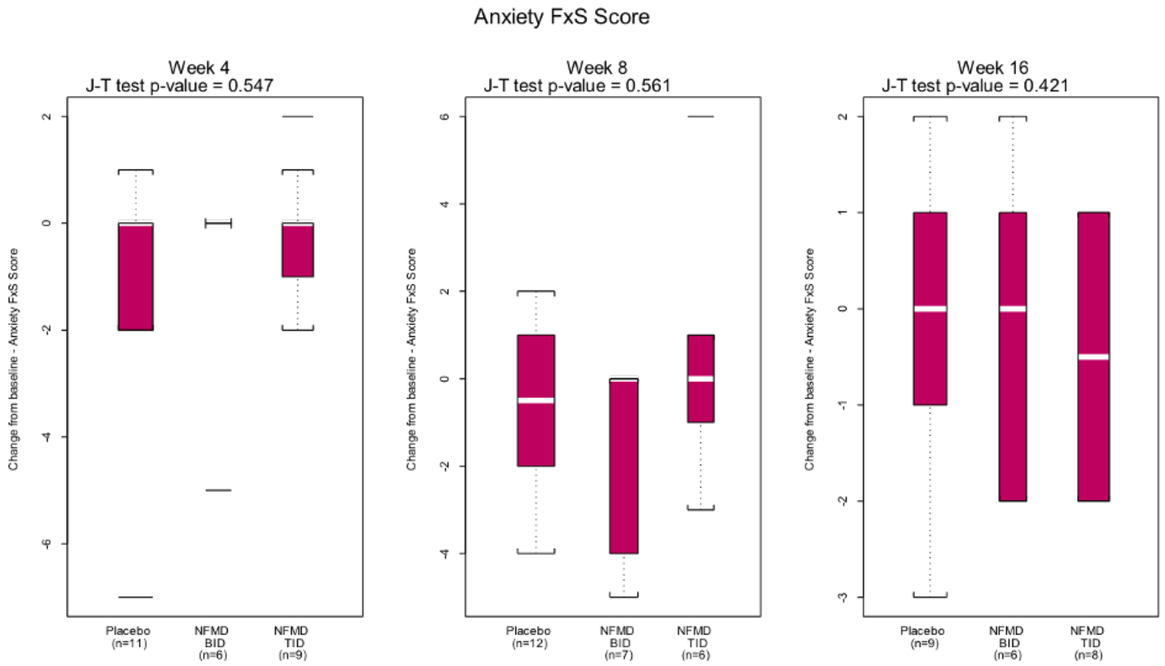

Supplementary Figure 3. Descriptive analyses of observed cognitive testing data in clinical study

a. **Neuropsychological Test Battery composite z-score** **b.** **Attention composite z-score**

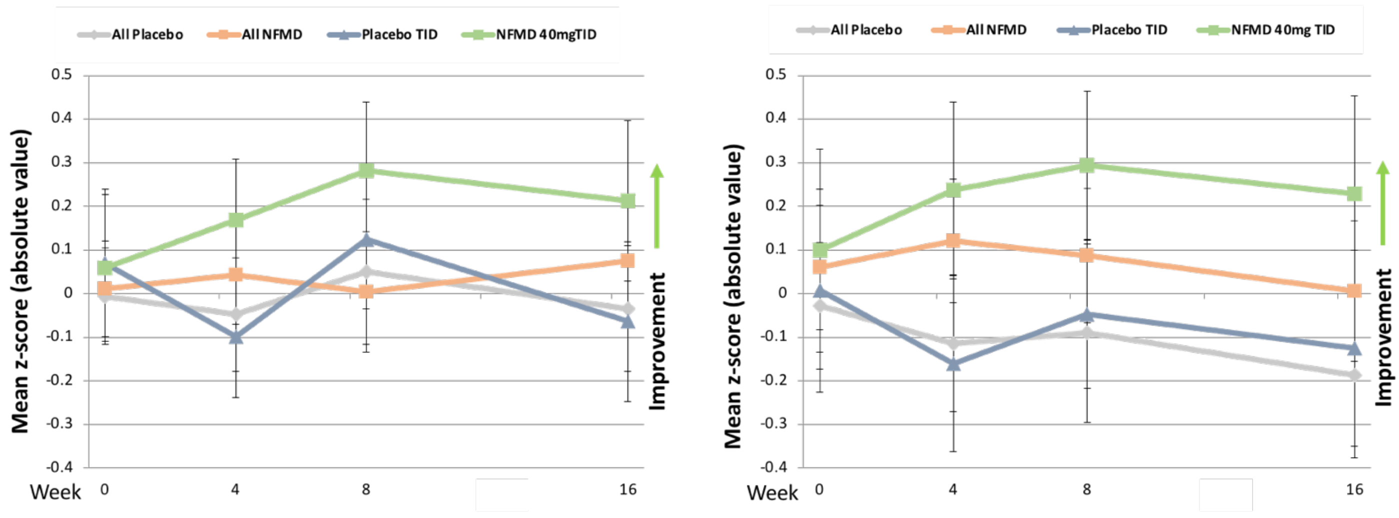

Number of Participants with Data at Each Timepoint (a)

Placebo	36	27	29
All NFMD	32	20	32
Placebo TID	23	17	15
NFMD TID	16	7	17

Number of Participants with Data at Each Timepoint (b)

Placebo	36	27	29
All NFMD	32	20	32
Placebo TID	23	17	15
NFMD TID	16	7	17

Legend.

a. Cognition, as assessed by a Neuropsychological Test Battery (NTB) composed of six tests that individually assess attention, executive function or visuospatial function. Mean (± SEM) absolute value of the composite z-score that includes results of all six tests are shown. b. Attention, as assessed by an Attention composite z-score that includes results on the two tests within the NTB that evaluate information processing speed, Detection and Identification. Mean (± SEM) absolute value of the attention composite z-score is shown. Because of Covid-19 lockdowns, not all study visits could take place onsite, and for all but the CDR-SB the assessments could only occur onsite, the number of participants, therefore, varies during the course of the study; the number of participants with data at indicated study visit are shown by treatment group below each outcome measure.

Supplementary Figure 4. Descriptive analyses of CDR-SB and TUG Test Results

a. Clinical Dementia Rating Sum-of-Boxes (CDR-SB)

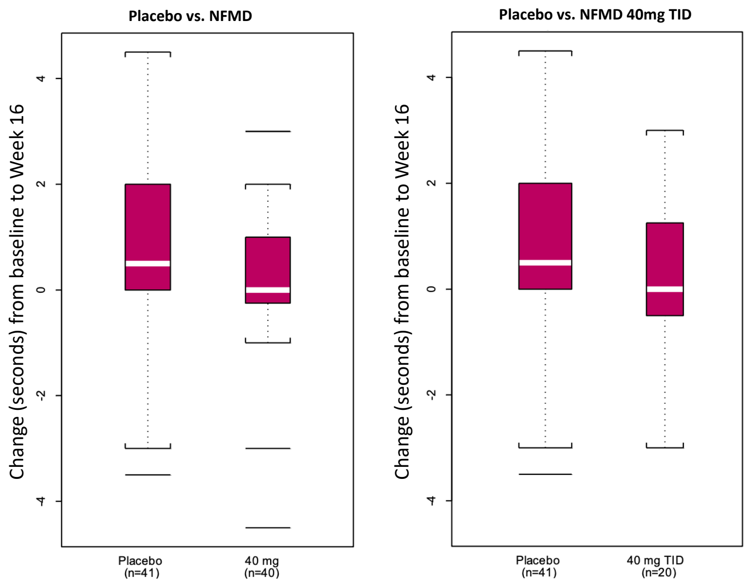

b. Timed Up and Go (TUG)

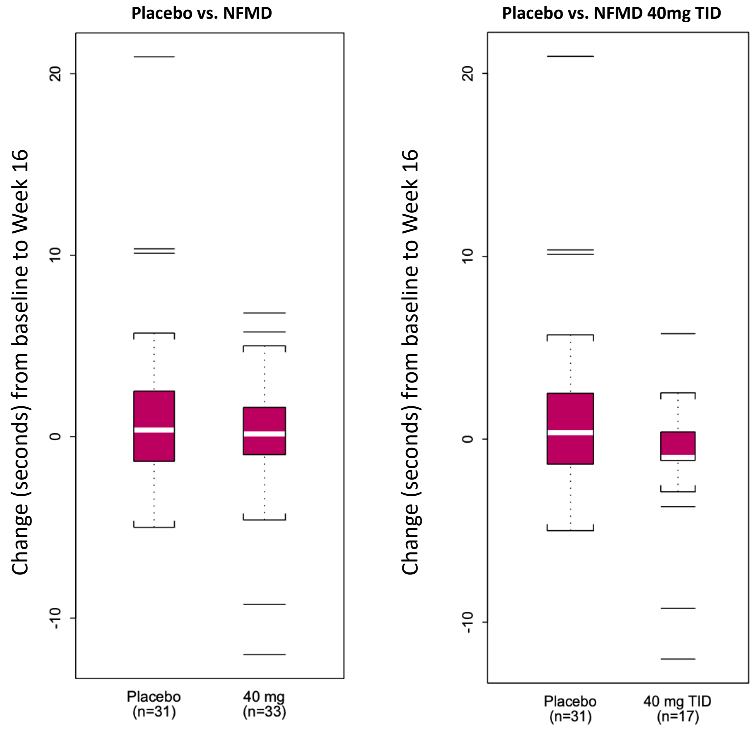

Legend. Change from baseline to week 16 [median, intra-quartile range in red, and range] is shown for each outcome measure. An increase in score reflects worsening.

Supplementary Figure 5. Change from Baseline on Individual Domains of the CDR-SB

a. Cognitive Domains

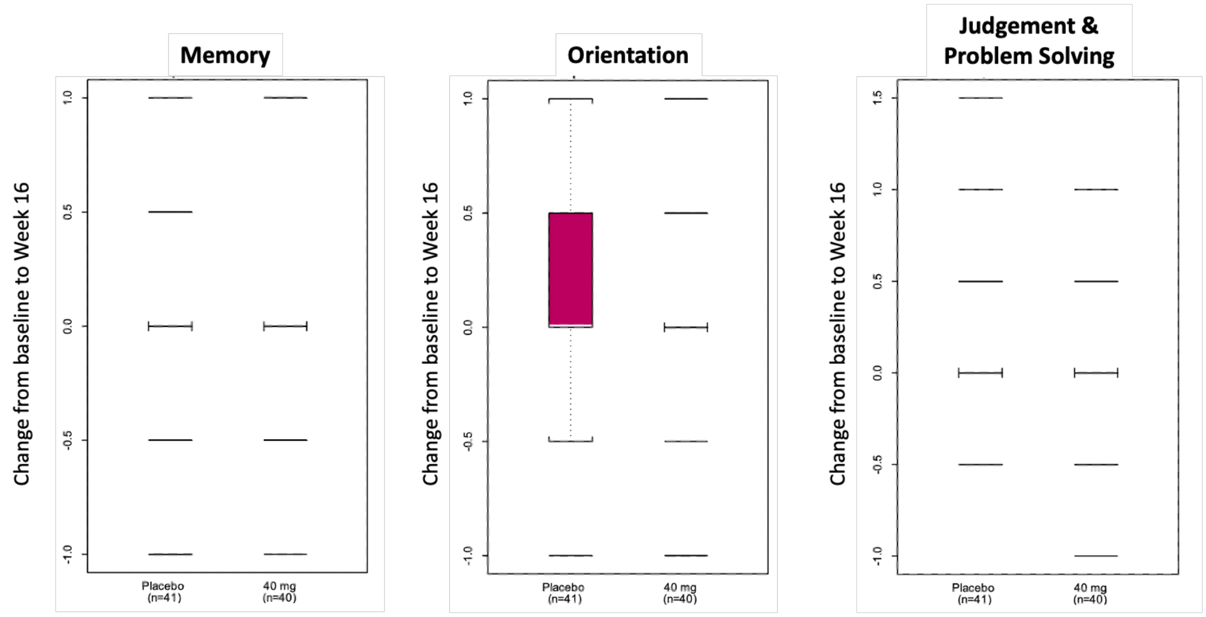

b. Functional Domains

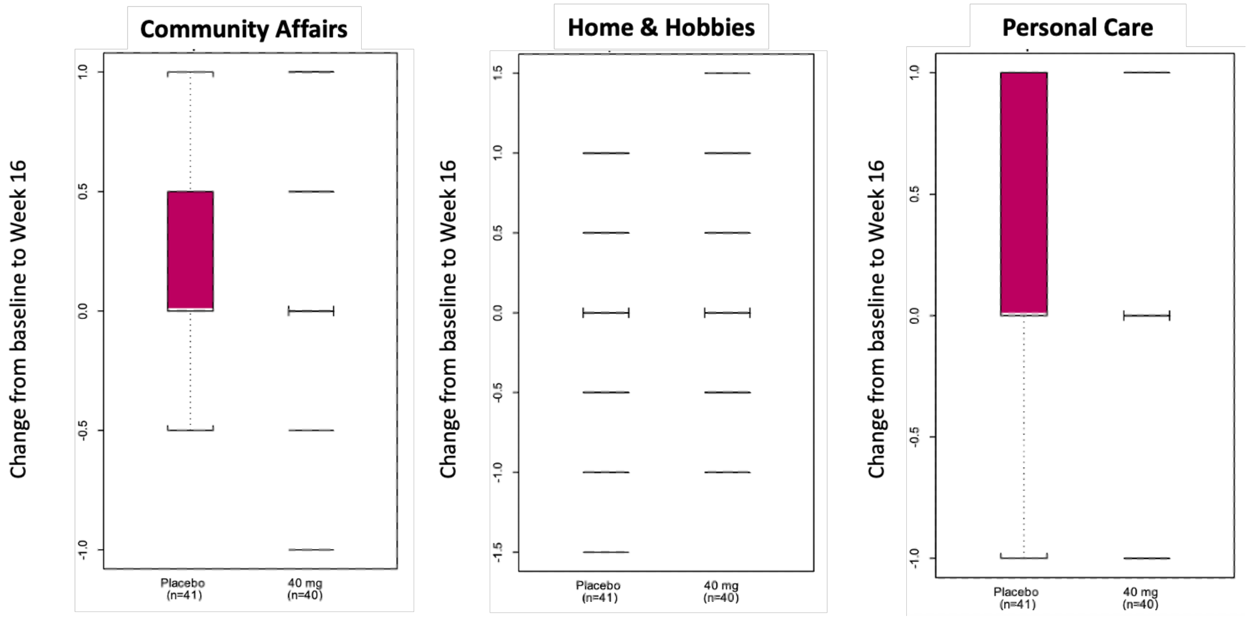

Legend. Change from baseline to week 16 [median, intra-quartile range in red, and range] is shown for each domain within the CDR-SB. An increase in score reflects worsening.

Supplementary Figure 6. Improvement in change from baseline between neflamapimod treatment and placebo (MMRM analysis of Change from Baseline) by baseline plasma ptau181 status

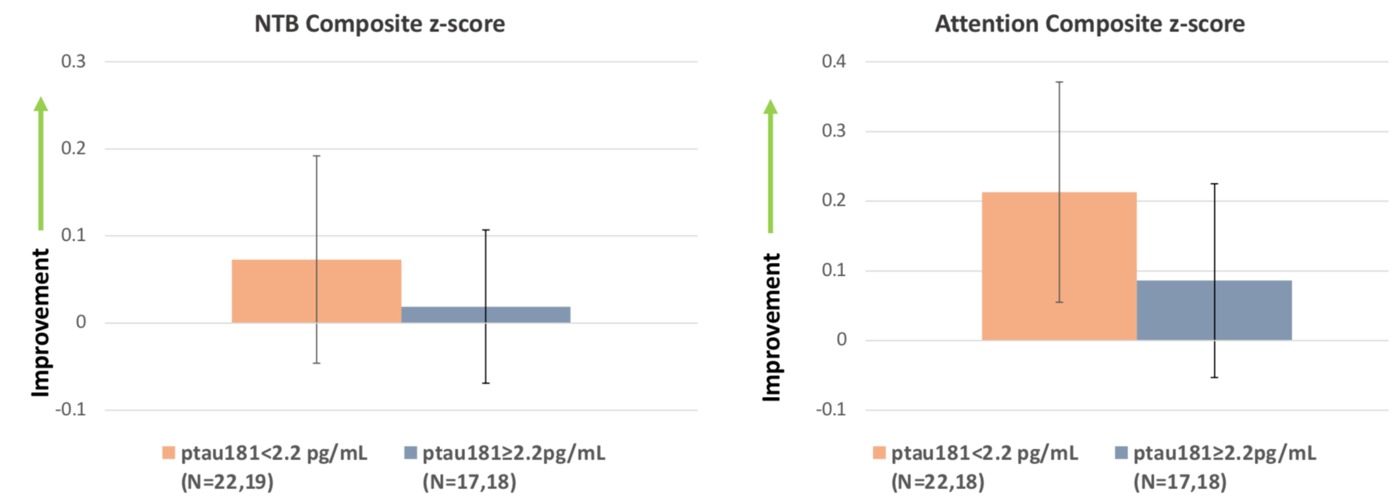

Baseline Scores: 0.11 (77) -.06 (.69) 0.08 (0.99) -0.08 (0.96)

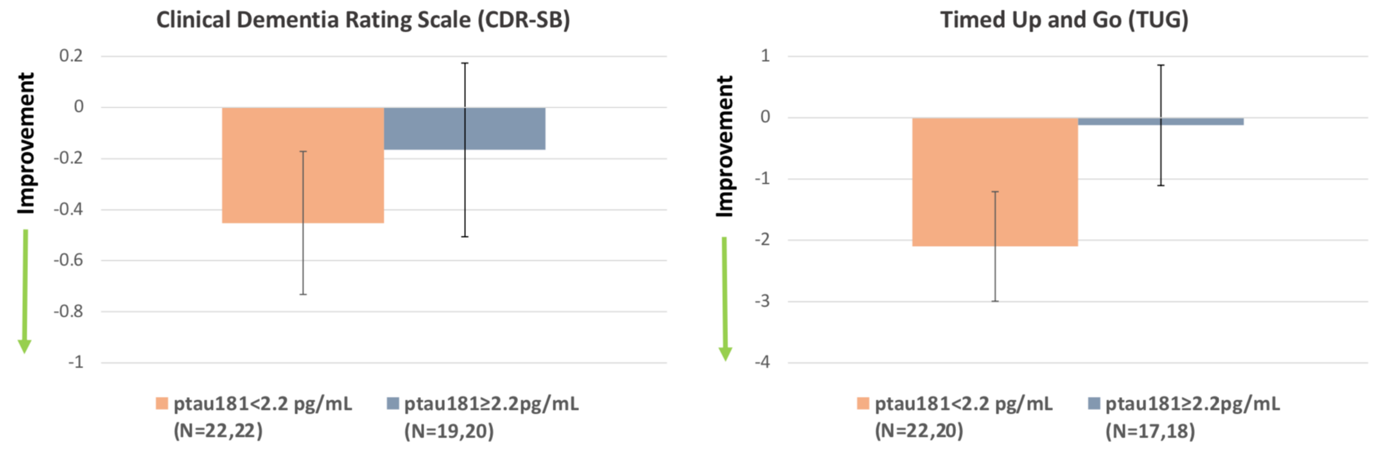

Baseline Scores: 4.6 (1.9) 5.7 (3.0) 12.1 (4.6) 13.4 (3.5)

Legend. Mean (+/- SEM) drug-placebo difference from MMRM analysis with baseline as a covariate is shown, stratified by baseline plasma ptau18 status (< or ≥ 2.2 pg/mL). The baseline [mean (SD)]score for each outcome measure by stratum is also provided. Number of participants denoted as N=, with first number representing number of neflamapimod participants and the second number representing placebo participants

Supplementary Table 1. Change from baseline MMSE scores on study by type of visit

	Treatment	Location	median	mean	N	SE
**MMSE**	All Placebo	On-site	-0.50	-0.56	60	0.311
		Remote	0.00	0.53	15	0.595
	All NFMD	On-site	-0.50	-0.74	55	0.329
		Remote	1.50	0.68	11	0.532
	Placebo TID	On-site	-0.50	-0.72	37	0.388
		Remote	0.25	0.75	10	0.876
	NFMD TID	On-site	-0.50	-0.52	26	0.437
		Remote	1.00	1.25	6	0.359

Supplementary Table 2. Serious Adverse Events (SAEs) Reported in the Study and Discontinuations due to Treatment Emergent Adverse Events (TEAEs)

Treatment Group	Number of SAEs	Descriptions (All considered not related to study drug)
Placebo BID	3	Hematochezia<br>Internal Bleeding<br>Intraparenchymal hemorrhage
Placebo TID	1	Asthma exacerbation
Neflamapimod 40mg BID	3	New brain lesions, consistent with brain metastasis (unknown primary)<br>Brain Tumor Diagnosis 34 days after last dose<br>Head Injury
Neflamapimod 40mg TID	0	-

Subjects Withdrawn due TEAE

	TEAE	Severity	Relationship to Study Drug
Neflamapimod 40mg BID	Brain lesion	Severe	Not Related
	Somnolence	Moderate	Possibly Related
	Head injury	Moderate	Not Related
Placebo BID	Hematochezia	Moderate	Not Related

Supplementary Table 3 MMRM Analysis of Clinical Outcome Measures – Neflamapimod 40mg TID vs. Placebo

	Number of Particpants		*Mean Baseline Values*		*Analysis of Change from Baseline*	
	NFMD 40mg TID	Placebo	NFMD 40mg TID	Placebo	Difference On-Study (95% CI)	Cohen's d Effect Size for Improvment - *d*
NTB Composite z-score*	19	37	0.06	0.07	0.17 (0.00, 0.35)	0.47
Attention Composite z-score	19	36	0.1	0.00	0.28 (0.04, 0.51)	0.41
Clinical Dementia Rating Sum of Boxes (CDR-SB)	20	42	4.7	5.1	-0.56 (-0.96,-0.16)	0.31
Timed Up and Go (TUG)	20	38	13.3	13.5	-1.4 (-2.6,-0.2)	0.50

*NTB: Neuropsychological Test Battery evaluating attention, executive function, and visual learning. Note: Difference (95% CI) from MMRM analysis. Improvement is reflected as increases in NTB and the Attention Compositte; and as decreases in CDR-SB and TUG test. Positive *d* indicates improvement relative to placebo, and negative *d* indicates worsening from baseline.

Supplementary Table 4 MMRM Analysis of Clinical Outcome Measures – Neflamapimod 40mg TID vs. Placebo TID

	Number of Particpants		*Mean Baseline Values*		*Analysis of Change from Baseline*	
	NFMD 40mg TID	Placebo TID	NFMD 40mg TID	Placebo TID	Difference On-Study (95% CI)	Cohen's d Effect Size for Improvment - *d*
NTB Composite z-score*	19	22	0.06	0.05	0.21 (0.00, 0.43)	0.49
Attention Composite z-score	19	23	0.09	-0.02	0.24 (-0.02, 0.51)	0.33
Clinical Dementia Rating Sum of Boxes (CDR-SB)	20	26	4.7	4.4	-0.63 (-1.06,-0.21)	0.38
Timed Up and Go (TUG)	20	23	13.3	13.3	-1.4 (-3.1,0.3)	0.44

*NTB: Neuropsychological Test Battery evaluating attention, executive function, and visual learning. Note: Difference (95% CI) from MMRM analysis. Improvement is reflected as increases in NTB and the Attention Compositte; and as decreases in CDR-SB and TUG test. Positive *d* indicates improvement relative to placebo, and negative *d* indicates worsening from baseline.
