# Supplementary material for: Preclinical and randomized clinical evaluation of the p38α kinase inhibitor neflamapimod for basal forebrain cholinergic degeneration
Source: Nat Commun. 2022 Sep 21;13:5308. doi: 10.1038/s41467-022-32944-3 (PMC9492778; doi:10.1038/s41467-022-32944-3)
Supplement: Supplementary file 3 — Reporting Summary [file 41467_2022_32944_MOESM3_ESM.pdf]

nature research

Corresponding author(s): John J. Alam, MD

Last updated by author(s): Aug 5, 2022

Reporting Summary

Nature Research wishes to improve the reproducibility of the work that we publish. This form provides structure for consistency and transparency in reporting. For further information on Nature Research policies, see our Editorial Policies and the Editorial Policy Checklist.

Statistics

For all statistical analyses, confirm that the following items are present in the figure legend, table legend, main text, or Methods section.

n/a	Confirmed	
☐	☒	The exact sample size (n) for each experimental group/condition, given as a discrete number and unit of measurement
☐	☒	A statement on whether measurements were taken from distinct samples or whether the same sample was measured repeatedly
☐	☒	The statistical test(s) used AND whether they are one- or two-sided<br>*Only common tests should be described solely by name; describe more complex techniques in the Methods section.*
☐	☒	A description of all covariates tested
☐	☒	A description of any assumptions or corrections, such as tests of normality and adjustment for multiple comparisons
☐	☒	A full description of the statistical parameters including central tendency (e.g. means) or other basic estimates (e.g. regression coefficient) AND variation (e.g. standard deviation) or associated estimates of uncertainty (e.g. confidence intervals)
☐	☒	For null hypothesis testing, the test statistic (e.g. F, t, r) with confidence intervals, effect sizes, degrees of freedom and P value noted<br>*Give P values as exact values whenever suitable.*
☒	☐	For Bayesian analysis, information on the choice of priors and Markov chain Monte Carlo settings
☒	☐	For hierarchical and complex designs, identification of the appropriate level for tests and full reporting of outcomes
☐	☒	Estimates of effect sizes (e.g. Cohen's d, Pearson's r), indicating how they were calculated

Our web collection on statistics for biologists contains articles on many of the points above.

Software and code

Policy information about availability of computer code

Data collection	Pre-clinical data were collected on to Excel (version 16.0) spreadsheets. The cognitive tests through Cogstate Ltd were collected via the web directly into an in-house electronic database at Cogstate. All other clinical data were collected through an in-house Electronic Data Capture (EDC) system at Cogstate.
Data analysis	The pre-clinical data were analyzed utilizing GraphPad version 8.0.1. Immunoblot band intensities were quantified with Fiji/ImageJ 2.3.0 (https://imagej.net/Fiji). Commercially available software [S-PLUS (Version 8.2), R (Version 3.6.3 or higher) or SAS (Version 9.3 or higher)] were used for the statistical analyses of the clinical study.

For manuscripts utilizing custom algorithms or software that are central to the research but not yet described in published literature, software must be made available to editors and reviewers. We strongly encourage code deposition in a community repository (e.g. GitHub). See the Nature Research guidelines for submitting code & software for further information.

Data

Policy information about availability of data

All manuscripts must include a data availability statement. This statement should provide the following information, where applicable:

- Accession codes, unique identifiers, or web links for publicly available datasets
- A list of figures that have associated raw data
- A description of any restrictions on data availability

The preclinical data associated with Figures 1-3 are provided in the Source Data file. Deidentified individual participant baseline data contained in Table 1 and on-study data from placebo recipients for the endpoints contained in Table 2 in this article will be made available upon reasonable request to the corresponding author (JJA) to investigators whose proposed use has been approved by an independent review committee, beginning 9 months, and ending 36 months after publication. The study protocol and statistical analysis plan (SAP) are available at https://clinicaltrials.gov/ct2/show/study/NCT04001517.

Field-specific reporting

Please select the one below that is the best fit for your research. If you are not sure, read the appropriate sections before making your selection.

☒ Life sciences ☐ Behavioural & social sciences ☐ Ecological, evolutionary & environmental sciences

For a reference copy of the document with all sections, see nature.com/documents/nr-reporting-summary-flat.pdf

Life sciences study design

All studies must disclose on these points even when the disclosure is negative.

Sample size	For the preclinical study No statistical methods were used to predetermine sample sizes, but our sample sizes are choices according to those reported in previous publications (Basavarajappa and Subbanna, Hippocampus 24:178-188; 2014; Jiang et al, Neurobiol Aging 39:8; Pensalfini et al, Cell Rep 33:108420, 2020). Based on the prior results, and preliminary data from a pilot study (n=3) a sample size of 9 per treatment group was selected for the main study. For the clinical study, no formal sample size calculation was performed, as this was a first study of neflamapimod in dementia with Lewy bodies and there was no good means to estimate treatment effect size. Instead, a samples size of 40 per group was chosen based on input from the cognitive testing vendor, Cogstate Ltd and their experience with the Cogstate battery. That experience indicated that 40 per group would be sufficient to meet the primary objective of the clinical study, that of evaluating the cognitive effects of neflamapimod in dementia with Lewy bodies.
Data exclusions	No data excluded from the analyses.
Replication	The preclinical study was partially replicated, as indicated in the discussion, an beneficial effect of neflamapimod on the number of cholinergic neurons in the medial septal nucleus was noted in the Rab5-overexpressing mouse (a different mouse model than that reported in the manuscript, and so not full replication). The clinical study is to be confirmed with a similar design study with larger numbers (n=80 per group, placebo TID vs. neflamapimod 40mg TID) once funding is obtained to conduct that study.
Randomization	Preclinical study allocation was by the animal handlers by separating into different cages for the different treatment groups by matching the age and sex as best as possible to reduce the possible variation . The clinical study randomization was accomplished utilizing a central Interactive Response Technology (IRT) system managed independently by Suvoda Inc (Conshohocken, PA). Randomization was stratified by ISLT Total Recall score at baseline (< 21 vs. > 21).
Blinding	In the preclinical study investigators conducting analyses (e.g. IHC) and collecting data (e.g. conducting LTP experiments) were blinded to treatment assignment. The clinical study was a fully double-blinded study with all participants, investigators and study staff, CRO staff, sponsor staff, etc. being blinded to treatment assignment until and after database lock (study investigators were unblinded to individual patient treatment assignments at study closeout).

Reporting for specific materials, systems and methods

We require information from authors about some types of materials, experimental systems and methods used in many studies. Here, indicate whether each material, system or method listed is relevant to your study. If you are not sure if a list item applies to your research, read the appropriate section before selecting a response.

Materials & experimental systems

n/a	Involved in the study	
☐	☒	Antibodies
☒	☐	Eukaryotic cell lines
☒	☐	Palaeontology and archaeology
☐	☒	Animals and other organisms
☐	☒	Human research participants
☐	☒	Clinical data
☒	☐	Dual use research of concern

Methods

n/a	Involved in the study	
☒	☐	ChIP-seq
☒	☐	Flow cytometry
☒	☐	MRI-based neuroimaging

Antibodies

Antibodies used	Immunohistochemistry was performed using commercial antibodies against Rab5-GTP (NewEast, 26911; 1:50), ChAT (Millipore Sigma; AB144; 1:250), Rab5 (Abcam; 18211; 1:1000) and visualized either with biotinylated (Vector Laboratories; 1:500) or fluorescence-conjugated secondary antibodies (Fisher Sci, 1:500). For protein analyses, western blot analyses were performed with antibodies against APP (c1/6.1; 1:1000), bCTF (M3.2, 1:250)12,66, BACE1 (Rockland; 200-401-984; 1:500), MAPKAPK-2 (MK2; Cell Signaling; 12155; 1:500), phospho-MK2 (Cell Signaling; 3007; 1:500), p38 MAPK (p38a; Cell Signaling; 9218; 1:500), phosphor-p38 (Santa Cruz; 166182; 1:500), MNK1 (Cell Signaling; 2195; 1:500), pMNK1 (Cell Signaling; 2111; 1:500), b-actin (Santa Cruz Biotechnology; sc-47778; 1:2000). All the secondary antibodies for Western blot analyses were from Jackson ImmunoResearch Laboratories and used 1:4000 dilution

	from 1 mg/ml stock solution. Diaminobenzidine (DAB) was visualized by incubating with biotinylated secondary antibody (1:500, Vector Laboratories) and Vectastain ABC kit (Vector Laboratories)." All the fluorescence-conjugated secondary antibodies (Fisher Sci) were used at 1:500 dilution.
Validation	Mouse monoclonal Anti-APP C-terminal clone ID C1/6.1 validated in multiple studies including Jiang et al. (2010) Proc Natl Acad Sci U.S.A. 107:12630-1635, PMID 20080541; http://antibodyregistry.org/AB_2715853<br><br>Rabbit monoclonal Anti-Rab5 (Abcam Cat. No. ab218624, http://antibodyregistry.org/AB_2892717)<br><br>Murine monoclonal Anti-Rab5-GTP (NewEast, 26911; http://antibodyregistry.org/AB_2262226)<br><br>Both Anti-Rab5 and Anti-Rab5-GTP antibodies had validated in multiple studies including Pensafini et al. (2020) Cell .Rep. 33:108420, PMID 33238112<br><br>Rabbit polyclonal Anti-p38 MAPK(p38; Cell Signaling; 9218), http://antibodyregistry.org/AB_10694846<br><br>Murine monoclonal Anti-phosphor-p38 (Santa Cruz; 166182), http://antibodyregistry.org/AB_2141746<br><br>Rabbit monoclonal Anti-MNK1 (Cell Signaling; 2195), http://antibodyregistry.org/AB_2235175<br><br>Rabbit polyclonal Anti-pMNK1 (Cell Signaling; 2111), http://antibodyregistry.org/AB_2266303<br><br>Rabbit monoclonal Anti-MAPKAPK-2 (MK2; Cell Signaling; 12155), http://antibodyregistry.org/AB_2797831 .<br><br>Rabbit monoclonal Anti-phospho-MK2 (Cell Signaling; 3007), http://antibodyregistry.org/AB_490936<br><br>Mouse monoclonal Anti-mouse beta C-terminal clone ID M3.2 validated in multiple studies including Jiang et al. (2016) Neurobio.Aging. 39:90-98, PMID 26923405<br><br>Rabbit polyclonal Anti-BACE1 (Rockland; 200-401-984), http://antibodyregistry.org/AB_2243187 ; Jiang et al. (2016) Neurobio.Aging. 39:90-98, PMID 26923405<br><br>Goat polyclonal Anti-ChAT antibody (Millipore;AB144); http://antibodyregistry.org/AB_90650 , Jiang et al. (2016) Neurobio.Aging. 39:90-98, PMID 26923405<br>Mouse monoclonal Anti-beta-actin (Santa Cruz Biotechnology; sc-47778; 1:2000), http://antibodyregistry.org/AB_626632

Animals and other organisms

Policy information about studies involving animals; ARRIVE guidelines recommended for reporting animal research

Laboratory animals	Ts2 (Stock No. 004850, n=33 female, 34 male) mice, and wild type breeding partner (B6EiC3SnF1/J, Stock No. 001875, n=32female, 36 male) from the same colony were obtained from The Jackson Laboratory (Bar Harbor, ME). Age predominantly 6-7 months (study on longitudinal decline in ChAT+ neurons included mice between 4 and 9.5 months of age). All the mice studied were taken care iof n the NKI Animal facility, kept 12 hours day and night cycle, at temperatures at approximately 70 ° Fahrenheit (plus or minus 2 °), and humidity kept between 40% and 60%.
Wild animals	No wild animals were used in the study
Field-collected samples	No field collected samples were used in the study
Ethics oversight	Mouse experimentation and animal care were approved by the Institutional Animal Care and Use Committee (IACUC) of the Nathan S. Kline Institute.

Note that full information on the approval of the study protocol must also be provided in the manuscript.

Human research participants

Policy information about studies involving human research participants

Population characteristics	Patients with mild-to-moderate DLB by consensus clinical criteria (McKeith et al, Neurology 89, 88-100 (2017including demonstrated abnormality in dopamine uptake by DaTscan™ (Ioflupane I123 SPECT), receiving cholinesterase inhibitor therapy (>3 months, stable dose > 6weeks) were included in the human clinical study. As this was by design a homogeneous patient population no baseline disease or demographic covariate was uitlized in the MMRM analysis (the one covariate was the baseline value of the endpoint being analyzed). Average age was 72.8 (range 59-87); 86% male, 14% female. Mean (SD) baseline Clinical Dementia Rating Sum of Boxes score was 5.0 (2.5).
Recruitment	As the great majority of clinical sites were academic sites at medical schools, patients were primarily recruited through the clinics affiliated with the respective institutions. Three centers, at which a total of ten patients were recruited, were independent clinical research centers that conduct pharmaceutical industry sponsored trials; in two of these center patients were recruited through advertising and reaching out to neurologists in the community (the third had a more direct affiliation to a neurology department at a local medical school). There is no reason to expect there was any bias in the selection of

	patients.
Ethics oversight	Conducted under FDA IND#125198 and a Clinical Trials Application with Centrale Commissie Mensgebonden Onderzoek (CCMO=Competent Authority) in the Netherlands. IRB/Ethics approvals provided by Copernicus Group IRB (CGIRB, Cary, NC), Western Institutional Review Board (WIRB, Puyallup, WA), Mayo Clinic Institutional Review Board (Rochester MN), Columbia University Medical Center Institutional Review Board (New York, NY), Cleveland Clinic IRB (Cleveland, OH), FoundationBeoordelingEthiekBiomedisch Onderzoek (BEBO, Assen, the Netherlands)

Note that full information on the approval of the study protocol must also be provided in the manuscript.

Clinical data

Policy information about clinical studies

All manuscripts should comply with the ICMJEguidelines for publication of clinical research and a completedCONSORT checklist must be included with all submissions.

Clinical trial registration	The trial was registered at clinicaltrials.gov as NCT04001517 and in the EU Clinical Trials Register with EudraCT Number of 2019-001566-15.
Study protocol	Study protocol and statistical analysis plan (SAP) available at https://clinicaltrials.gov/ct2/show/study/NCT04001517
Data collection	Study conducted at 24 centers, 22 in the US and 2 in the Netherlands (list of investtigators in the mansucript). Recruitment (randomization) took place between 30 September 2019 to 7 March 2020, and the last patient, last visit occurred on 14 July 2020
Outcomes	As a first study of neflamapimod in dementia with Lewy bodies, and as such was an exploratory study with no pre-defined the hypotheses. The primary objective was to evaluate the effect of neflamapimod on cognitive function as assessed in a study-specific Neuropsychological Test Battery (NTB) comprised of: Cogstate Detection test (DET), Cogstate Identification test (IDN), Cogstate One Card Learning test (OCL), Cogstate One Back test (ONB), Letter Fluency Test and Category Fluency Test (CFT). The secondary objectives of this study were to: (1) Evaluate the effects of neflamapimod on informant/caretaker evaluation of cognition and function, as assessed by the Clinical Dementia Rating Scale-Sum of Boxes (CDR-SB); (2) Assess the effects of neflamapimod on general cognition, as assessed by the Mini Mental State Examination (MMSE); (3) assess the effects of neflamapimod on episodic memory, as assessed by the International Shopping List Test (ISLT); (4) Assess the effects of neflamapimod on select domains of the 10-item Neuropsychiatric Inventory (NPI-10), including depression (dysphoria), anxiety, hallucinations, and agitation/aggression; (4) Evaluate the effects of neflamapimod on motor function as assessed by the Timed Up and Go Test (TUG); (5) Evaluate the effects of neflamapimod on quantitative electroencephalography (EEG). parameters.
